# Low temperature caused modifications in the arrangement of cell wall pectins due to changes of osmotic potential of cells of maize leaves (*Zea mays* L.)

**DOI:** 10.1007/s00709-016-0982-y

**Published:** 2016-05-19

**Authors:** Anna Bilska-Kos, Danuta Solecka, Aleksandra Dziewulska, Piotr Ochodzki, Maciej Jończyk, Henryk Bilski, Paweł Sowiński

**Affiliations:** 1 Department of Plant Biochemistry and Physiology, Plant Breeding and Acclimatization Institute - National Research Institute, Radzików, 05-870 Błonie, Poland; 2 Department of Plant Molecular Ecophysiology, Faculty of Biology, Institute of Plant Experimental Biology and Biotechnology, University of Warsaw, Miecznikowa 1, 02-096 Warsaw, Poland; 3 Department of Plant Pathology, Plant Breeding and Acclimatization Institute - National Research Institute, Radzików, 05-870 Błonie, Poland; 4 Laboratory of Electron Microscopy, Nencki Institute of Experimental Biology, PAS, Pasteura 3, 02-093 Warsaw, Poland; 5 Department of Plant Physiology, Institute of Applied Biotechnology and Basic Science, University of Rzeszow, Werynia 502, 36-100 Kolbuszowa, Poland; 6 Biomedical Sciences Research Complex North Haugh, University of St. Andrews, KY16 9ST, St. Andrews, Fife, Scotland, UK

**Keywords:** Cell wall, Cold stress, Osmotic potential, Pectins, *Zea mays*

## Abstract

The cell wall emerged as one of the important structures in plant stress responses. To investigate the effect of cold on the cell wall properties, the content and localization of pectins and pectin methylesterase (PME) activity, were studied in two maize inbred lines characterized by different sensitivity to cold. Low temperature (14/12 °C) caused a reduction of pectin content and PME activity in leaves of chilling-sensitive maize line, especially after prolonged treatment (28 h and 7 days). Furthermore, immunocytohistological studies, using JIM5 and JIM7 antibodies, revealed a decrease of labeling of both low- and high-methylesterified pectins in this maize line. The osmotic potential, quantified by means of incipient plasmolysis was lower in several types of cells of chilling-sensitive maize line which was correlated with the accumulation of sucrose. These studies present new finding on the effect of cold stress on the cell wall properties in conjunction with changes in the osmotic potential of maize leaf cells.

## Introduction

Cell walls, “the first line of protection”, take a crucial part in the plant functioning, starting from determination of cell shape, due to its mechanical properties, to the protection against negative impact of environmental factors. Dynamic nature of the cell wall is maintained by modification of polysaccharides – its main structural component. In general, primary cell wall polysaccharides in the majority of terrestrial plants can be classified into three groups: cellulose, hemicelluloses, and pectins where cellulose is organized in microfibrils embedded in the matrix formed by pectins and hemicelluloses (Park and Cosgrove 2015). Pectins characterized by high galacturonic acid content, were classified into four main groups: homogalacturonan (HGA), rhamnogalacturonan I (RGI), rhamnogalacturonan II (RGII), and xylogalacturonan (XGA) (Ridley et al. 2001; Mohnen 2008). HGA polysaccharide, the major compounds of pectin, consists of galacturonosyl residues which may be methylesterified, acetylated and/or substituted with other subunits, including xylose (Schols et al. 1995; O’Neill and York 2003). Chemical modifications in HGA chains, especially the process of de-esterification (and subsequent calcium binding), which is under the control of pectin methylesterase (PME, EC. 3.1.1.11) have an influence on the biomechanical properties (e.g., plasticity, stiffness) of the cell wall (Parre and Geitmann 2005; Siedlecka et al. 2008; Yang et al. 2010; Hongo et al. 2012; Liu et al. 2013). Furthermore, the level of methyl esterification of pectins can indicate the stage of actual growth and development of cells (Willats et al. 2001). The structure and composition of cell wall can be modified by various abiotic stress factors. Changes may affect the biomechanical properties, e.g., through chemical modifications of the cell wall components. For instance, in coffee (*Coffea arabica* L.) leaves salt stress caused changes in pectic fractions, which led to the stiffening of the cell wall resulting in decreasing of its permeability for salt (Lima et al. 2014). Similarly, in petioles of an aspen hybrid (*Populus tremula × tremuloides*) increased rigidity of the cell wall formed a barrier for salt entrance (Muszyńska et al. 2014). Drought induced changes in the concentration of hemicellulose in switchgrass (*Panicum virgatum* L.) (Jiang et al. 2012). Next, phenomenon of desiccation tolerance of xerophyte plant (*Myrothamnus flabellifolius*) is connected with arabinan-rich leaf cell walls (Moore et al. 2006). Whereas, the acclimation of maize (*Zea mays*) root to drought is associated with the increase of wall-linked phenolics in the tip and lignins in the stele (Fan et al. 2006). Deficit in the mineral nutrition led to reduction of cellulose content and a lower degree of pectin methyl esterification in grapevine (*Vitis vinifera* L.) callus (Fernandes et al. 2013). Aluminum stress caused an increase in the content of pectin in roots of maize and wheat, while in cell wall of flax hypocotyl (*Linum usitatissimum* L.) treated with cadmium content of high-esterified homogalacturonans were reduced (Eticha et al. 2005; Hossain et al. 2006; Douchiche et al. 2010). Overexpression of enzyme responsible for pectin degradation (the *β* subunit of polygalacturonase 1) led to increased sensitivity of transgenic rice plants to cold, drought, and salt stresses (Liu et al. 2014). High temperature (37 °C) induced arabinose and galactose overaccumulation and reduced the mannose, glucose, uronic acid, rhamnose, and fucose contents in coffee leaves (Lima et al. 2013). Solecka et al. (2008) demonstrated that in cold-acclimated leaves of winter oil-seed rape changes in the pectins content and PME activity have been associated with modifications of the biomechanical properties (higher rigidity) of cell wall. Cold acclimation also resulted in increase in the content of other cell wall sugars, such as galactose, arabinose, and glucose in these plants (Kubacka-Zębalska and Kacperska 1999). In response to cold acclimation of C_4_ grass from genus *Miscanthus*, a significant increase in phenylalanine ammonia-lyase (PAL) activity and decrease in lignin content in cell walls were observed (Domon et al. 2013).

Specific regions of cell wall, called “pit fields”, are traversed by plasmodesmata, minute channels by which the intercellular transport occurs in plants. These structures are particularly important for C_4_ plants where the photosynthesis process is distributed spatially. In more detailed view: in the mesophyll cells primary carbon assimilation (PCA) occurs while associated process of the photosynthetic carbon reduction (PCR) is carried out in the bundle sheath cells (Hattersley 1984). This spatial distribution of photosynthesis requires an efficient intercellular transport and thus, the sufficient permeability of plasmodesmata. It is also possible that the capacity of plasmodesmata may depend on the properties of surrounding cell walls.

This work was aimed on verifying the hypothesis that the decrease in osmotic potential of cells of maize leaves at cold through the sucrose accumulation may be associated with changes in cell wall properties, specified by pectin reorganization. This hypothesis has been emerged from our earlier work on changes of transcriptome in leaves of maize, where numerous genes related to cell wall functioning responded to cold stress (Trzcinska-Danielewicz et al. 2009; Sobkowiak et al. 2014). It may suggest that chilling treatment modifies the cell wall properties. Additionally, our previous studies indicated that low temperature inhibited intercellular transport in leaves of chilling-sensitive maize line what was related to the closure of plasmodesmata on the photosynthetic pathway (Bilska and Sowiński 2010). Thus, in this work particular attention has been paid to regions of cell wall with plasmodesmata (pit fields). Modifications of plasmodesmata ultrastructure could lead to increase of sucrose content in photosynthetically active cells and changes in their osmotic potential what could be stimulus for changes of the cell wall properties.

## Materials and methods

### Plant material, growth condition and chilling-treatment periods

For studies chilling-tolerant (CT) KW 1074 (*Zea mays* spp. indurata, flint) and chilling-sensitive (CS) CM 109 (Z. *mays* spp. indentata, dent) maize lines were used. Differences in the chilling sensitivity of the inbred lines used have been described elsewhere (Sowiński 1995). Kernels were germinated in wet sand in darkness at 25 °C. Then, plants were transferred to hydroponic media (Knop solution supplemented with Hoagland’s micronutrients). Seedlings were grown in a growth chamber with parameters set to: 14/10 h light/darkness, irradiance 250 μmol quanta m^−2^ s^−1^ at 24/22 °C (day/night temperature). When the third leaf was fully developed, at the beginning of the light period, plants were transferred to low temperature 14/12 °C (day/night) for either 1, 4, 28, or 168 h (7 days). Chilling treatment was started at the beginning of the light period and control samples were taken 4 h after the light had been switched on, except the analysis of the sucrose content, where additional control (variants: c0, c1, c4, c8, c12, c28) and chilled (1, 4, 8, 12, and 28 h) plants were used. Each analysis was repeated three times in four independent experiments.

### Cell wall preparation and pectin content determination

The analysis of pectin content, PME activity and pectin immunolocalization was performed for control plants and those chilled for 4 h, 28 h, and 7 days. Cell walls from maize leaf laminas were prepared using a modified method of Wu et al. (1996). Fresh leaf tissues were homogenized at 4 °C in HEPES buffer (0.05 M, pH 6.8), containing a mixture of protease inhibitors (PMSF, aprotinin, bestatin, pepstatin A, and leupeptin), filtered through a miracloth and washed several times with cold water. After air drying, crude cell wall preparations from maize leaves were weighted and used for determination of pectin content. Cell wall content was expressed in milligrams per 1 g of leaf dry weight (DW).

Pectin isolation was performed as described by Kubacka-Zębalska and Kacperska (1999). In brief: crude cell wall preparations were subjected to 90 % DMSO treatment to remove starch.

The Lugol’s test was used to confirm that the material is free of starch (data not shown).

Air-dried cell wall aliquots (1 g) were extracted with a mixture of CDTA and Na-acetate (50 mM, pH 6.5), for 6 h and then with CDTA (50 mM) for 2 h at room temperature. The combined extracts were centrifuged (12,000 g, 15 min) and concentrated by evaporation under vacuum. The concentrate was dialyzed for 72 h against deionized water and dried under vacuum. Pectin content was expressed in milligrams per 1 gram of cell wall preparations. Differences between the experimental variants were evaluated by Tukey test at 0.05 and 0.01 probability levels, using STATISTICA 7.0 PL software (Statsoft, USA).

### Determination of PME activity

Determination of the enzymatic activity was performed according to Solecka et al. (2008). Cell wall proteins were extracted from crude cell wall preparations with HEPES buffer (0.05 M, pH 6.8), containing 1 M NaCl and a mixture of protease inhibitors (PMSF, aprotinin, bestatin, pepstatin A, and leupeptin). Protein concentration in extracts was determined by the Bradford method (1976), using bovine serum albumin (Sigma, Germany) as a standard. Protein extracts from cell walls were used for determination of PME activity.

Reaction mixtures contained 0.5 % (*w*/*v*) highly-methylated citrus pectins (Sigma, Germany), 0.2 M NaCl and 0.015 (*w*/*v*) Methyl Red (Sigma, Germany) as a pH indicator. Changes in color from yellow to red (due to pH lowering during pectin de-esterification) were measured spectrophotometrically at 525 nm (Shimadzu, Japan) for 3 min at 25 °C. A calibration curve was obtained by adding 1 to 200 nEq H+ to 1 ml of reaction mixture and the enzyme activity was calculated on the basis of cell wall proteins. The enzymatic activity was expressed in PME units. One unit was defined as one nanoequivalent of protons (nEH^+^) released by one milligram of cell wall proteins during 1 min, and was also recalculated per gram of cell wall matter. Differences between the experimental variants were evaluated by Tukey test at 0.05 and 0.01 probability levels, using STATISTICA 7.0 PL software (Statsoft, USA).

In preliminary experiments, the PME activity was measured at three different pH values: 5.0, 6.8, and 8.5. PME activities determined at acidic or alkaline pH were low and were not significantly affected by the cold-acclimation conditions (data not shown). Therefore, only the enzymatic activities determined at neutral pH are reported in the present paper.

### Immunolocalization of pectins

For these studies samples were fixed in 4 % paraformaldehyde (PFA) in 0.1 M phosphate buffer, pH 7.3 at room temperature. After dehydration (ethanol 10–100 %) the material was embedded in LR White resin (London Resin Company, UK) and polymerized for 24 h at 37 °C. Ultrathin (80 nm) sections were cut with a diamond knife on a Leica Ultracut UTC ultramicrotome. Immunolocalization of pectins were performed using monoclonal antibodies (Plant Probes, UK) against low-esterified pectins (rat IgG, JIM5) and against highly esterified pectins (rat IgA, JIM7) (Knox et al. 1990; Clausen et al. 2003). In the first stage of immunolabeling, the blocking of unspecific epitopes was performed using powdered milk solution (0.3 %, *w*/*v*) with Tween (0.1 %) in PBS (0.1 M, pH 7.3). After series of washing cycles (in PBS, 0.1 M, pH 7.3), samples were incubated for 1.5 h at room temperature with primary antibodies (JIM5 and JIM7)—both diluted 1:100. In case of negative control this step was omitted. Next, the material was washed (PBS, 0.1 M, pH 7.3) and incubated for 1 h at room temperature with secondary antibodies conjugated to 10 nm gold particles (goat anti-rat, SIGMA) in 1:30 dilution. Samples were finally contrasted with uranyl acetate for 20 min (5 %) and lead citrate for 30 min (0.04 %). Electron microscope observations were done using transmission electron microscope (model JEM 1200 EX; JEOL Co., Japan).

### Quantitative analysis of labeling density

The quantitative analysis of labeling density was performed following the method described by Morvan et al. (1998) using iTEM software (Olympus, Germany). Gold particles were counted from cell wall section (at least 30 sections per experimental variant) at each analyzed interface. The values of labeling density are reported as the mean numbers of gold particles per 1 μm^2^. For the analysis, six different cellular interfaces were chosen (Table 1). Gold particles from the cell wall forming intercellular spaces were not taken for counting. The differences, as calculated by comparing the number of gold particles between control (unchilled) and chilled leaves for each cellular interface were analyzed using the Kruskal-Wallis test with Bonferroni correction procedure (SAS 9.1.3; SAS Institute, Cary, NC).

**Table 1:** Types of cellular interfaces selected for the quantitative analysis of labeling density

Cellular interface	Abbreviation
Kranz mesophyll/bundle sheath	KMS/BS
Bundle sheath/vascular parenchyma	BS/VP
Vascular parenchyma/companion cell	VP/CC
Vascular parenchyma/thin-walled sieve	VP/SE
Vascular parenchyma/thick-walled sieve	VP/SET
Companion cell/thin-walled sieve	CC/SE

For this analysis at least 30 cell wall sections (with an area of 1 μm^2^) per experimental variant were taken

### Sucrose content

The analysis of sucrose content was performed for control plants—variants: c0, c1, c4, c8, c12, and c28—and plants chilled for 1, 4, 8, 12, and 28 h using gas chromatography according to method described by Knudsen and Li (1991). Briefly, the fragment of leaf (about 200 mg) was crushed in liquid nitrogen. Next, the samples were homogenized using Ultra-Turrax 25 (IKA—Werke GmbH & Co. KG, Germany) with the addition of the internal standard: phenyl-β-_D_-glucopyranoside (Sigma-Aldrich). After evaporation, the precipitate was dissolved by heating in hydroxylamine hydrochloride in pyridine (25 mg/ml). Next, derivatization was performed with hexamethyldisilazane (Sigma-Aldrich) and trifluoroacetic acid. Samples were analyzed using gas chromatograph HP 5890 Series IIA (Chemstation) equipped with a flame ionization detector (FID) and a quartz capillary column (30 m × 0.53 mm). The chromatograph was connected to an autosampler HP 7363 (Hewlett-Packard) and controlled by Chemstation workstation.

### Plasmolysis

Plasmolysis was determined for control plants and plants chilled for 1 and 4 h using a graded series of sorbitol solutions ranging from 0 to 1400 mM, in steps of 100 mM, as described elsewhere (Turgeon and Medville 1998). The method of the incipient plasmolysis allows studying directly the osmotic pressure of individual cells (osmoticum concentration of 1 M equals to 2.78 MPa of osmotic pressure, Turgeon and Medville 1998). For this approach, leaf samples were first pre-incubated for 20 min in an appropriate sorbitol solution (in 25 mM PIPES-NaOH buffer) and then fixed overnight at 4 °C in 2.5 % glutaraldehyde supplemented with sorbitol at a concentration as during the pre-incubation. Next, samples were washed three times in PIPES-NaOH buffer and were post-fixed in 1 % osmium tetroxide for 2 h at room temperature. After dehydration (ethanol 10–100 %) the material was embedded in the epoxy resin (Epon, Serva). It was assumed that the plasmolysis occurs when more than 30 % of plasmalemma in the cell of a given type separated from the cell wall.

## Results

### Changes in the cell wall and pectin content in leaves of maize due to chilling treatment

**Fig. 1:**
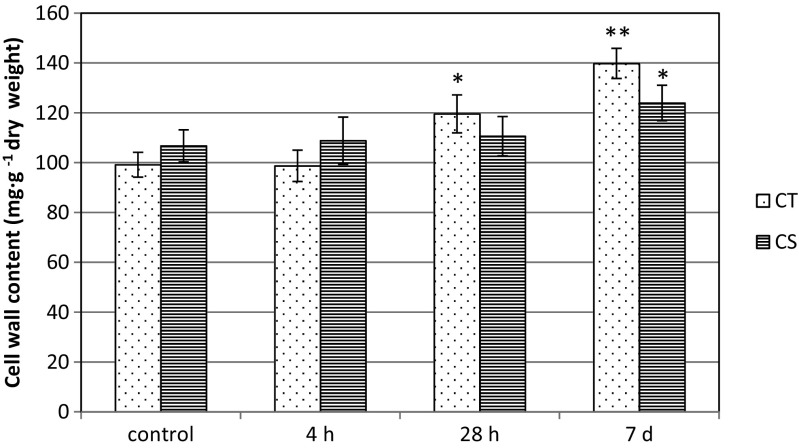
Cell wall content in leaf cells of two maize inbred lines: chilling-tolerant (*CT*) and chilling-sensitive (*CS*) treated with low temperature for three time periods: 4 h, 28 h, and 7 days. *Bars* represent the mean ± SD values. *P < 0.05, **P < 0.01

**Fig. 2:**
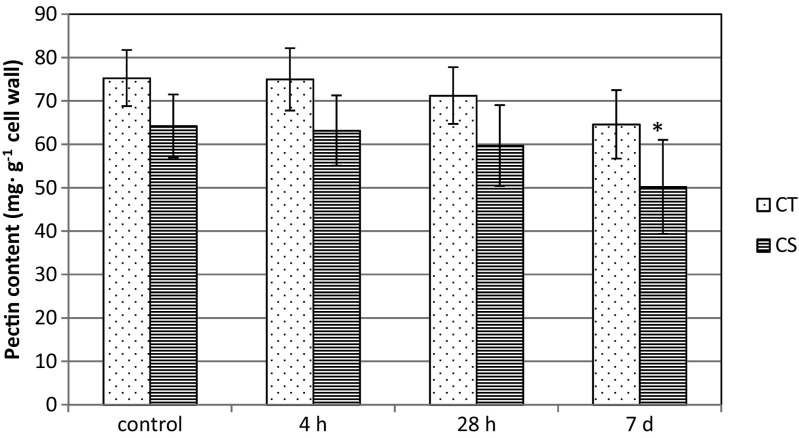
Global pectin level in cell wall of leaf cells of two maize inbred lines: chilling-tolerant (*CT*) and chilling-sensitive (*CS*) treated with low temperature for three time periods: 4 h, 28 h, and 7 days. *Bars* represent the mean ± SD values. *P < 0.05, **P < 0.01

In order to investigate the effects of chilling treatment on the cell wall properties of maize leaves the cell wall content as well as the pectin content and PME activity were determined. In this approach, the first step was the analysis of the cell wall content in leaf. The level of the cell wall fraction was similar in control plants of tested maize lines and was about 99 and 107 (mg g^−1^ dry weight), for chilling-tolerant (CT) and chilling-sensitive (CS) lines, respectively (Fig. 1). Comparing to control in cold the content of cell wall fraction was higher in leaves of both maize lines. The effect was particularly significant in the CT line, where an increase of about 40 % after prolonged chilling (7 days) was noted (Fig. 1). A marked decrease of pectin content was observed in chilled leaves of CS line after the week-long cold treatment (Fig. 2).

### The influence of low temperature on pectin methylesterase activity in leaves of maize

**Fig. 3:**
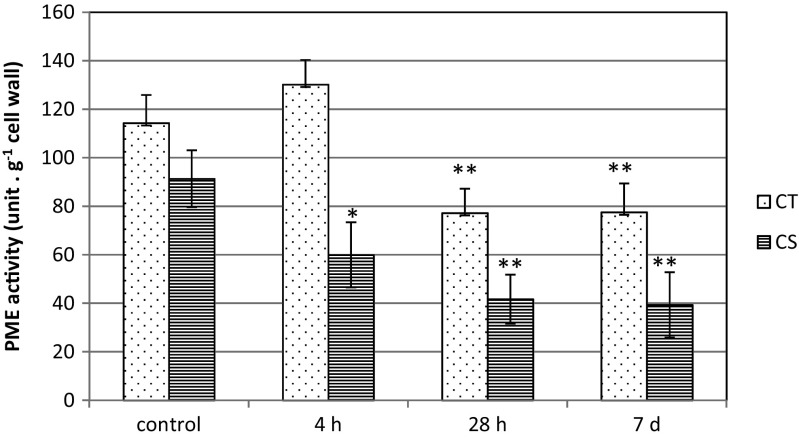
Pectin methylesterase activity in leaf cells of two maize inbred lines: chilling-tolerant (*CT*) and chilling-sensitive (*CS*) treated with low temperature for three time periods: 4 h, 28 h and 7 days. Bars represent the mean ± SD values. *P < 0.05, **P < 0.01

In CT line, at first a slight increase in PME activity was observed followed by the decrease after prolonged chilling treatment: 28 h and 7 days (Fig. 3). In contrast, in leaves of CS line, PME activity significantly decreased just after 4 h of chilling treatment and was about 35 % lower compared to leaves of control plants (Fig. 3). These changes intensified along with lengthening of chilling-treatment period—up to about 55 % in comparison to control. On the contrary, the decrease in PME activity in CT leaves observed after prolonged chilling treatment was only about 30 % (Fig. 3).

### Pectin immunolocalization in cell wall—microscopic observations

**Fig. 4:**
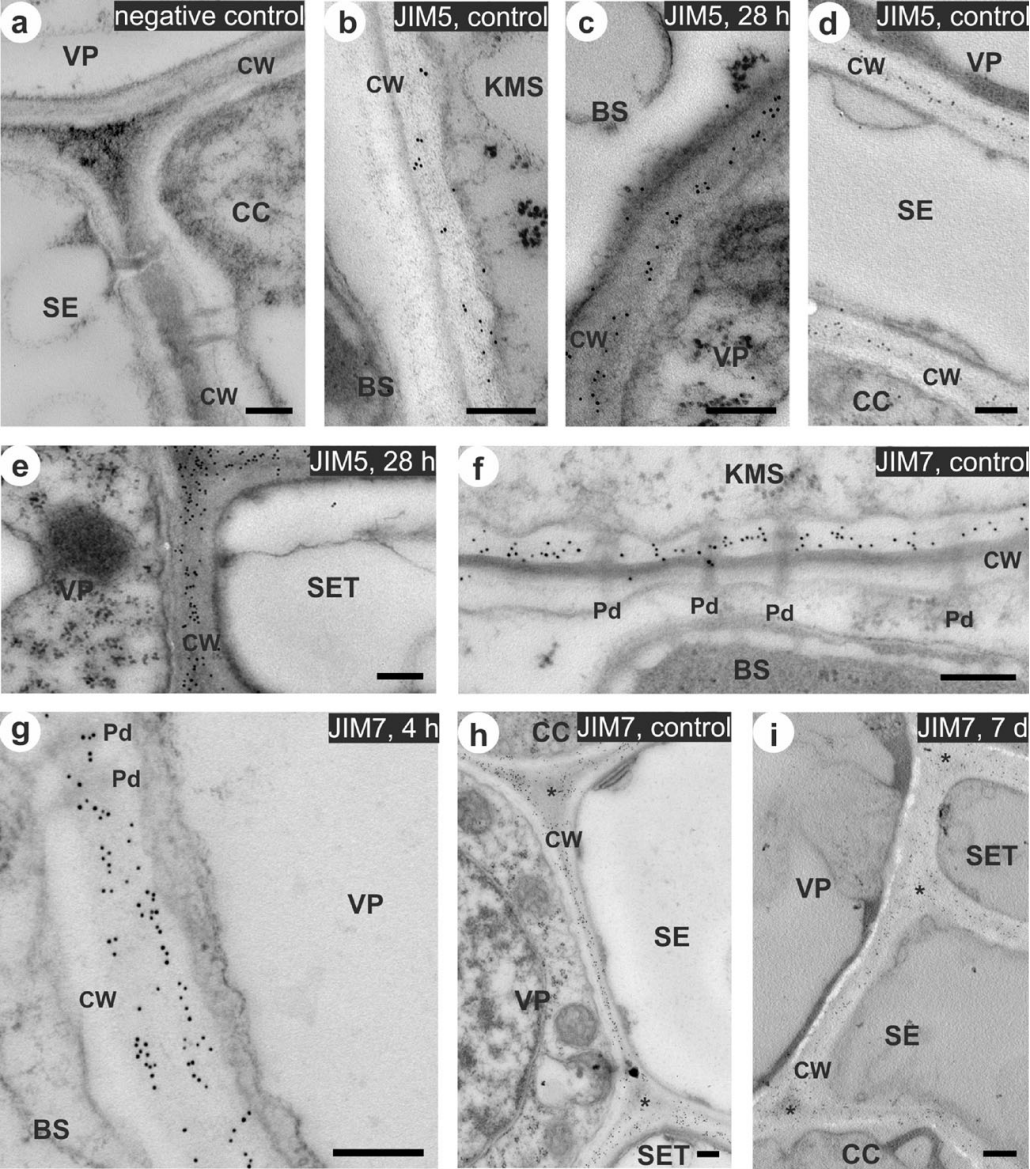
Immunogold localization of pectins using monoclonal antibodies: JIM5 specific to low-esterified pectins (**b–e**) and JIM7 specific to high-esterified pectins (**f–i**) in leaves of chilling-tolerant maize line. Negative control, where the incubation with primary antibodies was omitted (**a**). Control (non-chilled) leaves (**b**, **d**, **f**, **h**) and chilled leaves for 4 h (**g**), 28 h (**c**, **e**) and 7 days (**i**). Gold particles were mainly observed in the primary cell wall close to the middle lamella (**b–f**), with no relationship with the presence of plasmodesmata (**f**, **g**), and in the intercellular spaces (**h**, **i**). In chilled leaves, for VP/SE and CC/SE interfaces decrease in the density of labeling was observed (**h**, **i**). *KMS* Kranz mesophyll; *BS* bundle sheath; *VP* vascular parenchyma; *CC* companion cell; *SE* thin-walled sieve tube; *SET* thick-walled sieve tube; *CW* cell wall; *Pd* plasmodesmata; *intercellular space. *Scale bar*, 200 nm

**Fig. 5:**
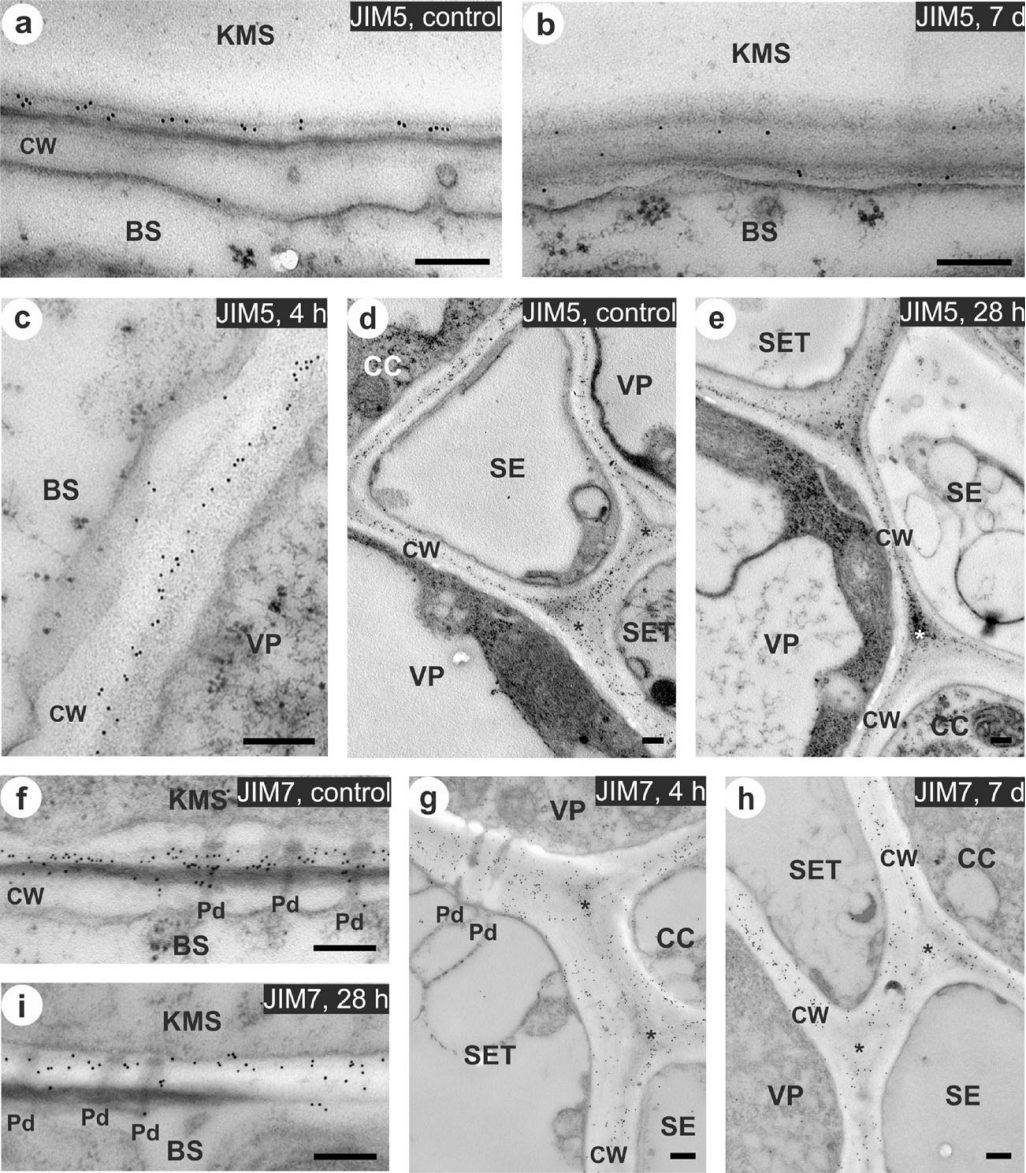
Immunogold localization of pectins using monoclonal antibodies: JIM5 specific to low-esterified pectins (**a–e**) and JIM7 specific to high-esterified pectins (**f–i**) in leaves of chilling-sensitive maize line. Control (non-chilled) leaves (**a**, **d**, **f**) and chilled leaves for 4 h (**c**, **g**), 28 h (**e**, **i**), and 7 days (**b**, **h**). Chilling caused reduction of number of gold particles, especially for: KMS/BS interface (**a**, **b**, **f**, **i**), VP/SET and CC/SE interfaces (**d**, **e**, **g**, **h**). *KMS* Kranz mesophyll; *BS* bundle sheath; *VP* vascular parenchyma; *CC* companion cell; *SE* thin-walled sieve tube; *SET* thick-walled sieve tube; *CW* cell wall; *Pd* plasmodesmata; *intercellular space. *Scale bar*, 200 nm

The observed cell system in the vascular vein, vein size, and distribution of veins in the leaf tissue were consistent with the typical leaf anatomy of C_4_ plants. The cell wall of bundle sheath cells was characterized by suberin layer which was observed in the middle lamella (Evert et al. 1996). Signal coming from both types of antibodies—JIM5 and JIM7, was detected in cell walls of all of the examined cellular interfaces (Fig. 4, and 5). Negative control where primary antibodies were omitted confirmed the reliability of the method – the labeling was not found within cell wall as well as in other cellular localization (Fig. 4a).

In most cell types of studied maize lines, pectins recognized by both types of antibodies were observed predominantly in the primary cell wall close to the middle lamella (Fig. 4b–f, h Fig. 5c–f), as well as in the intercellular spaces (Fig. 4h, i, Fig. 5d, e, g, h). In case of all interfaces the distribution of gold particles was not dependent on the plasmodesmata presence (Fig. 4f, g, Fig. 5f, i). Chilling treatment did not cause significant changes in the intensity of labeling in the leaves of CT line. In contrast, plants of CS line showed in most cases reduction in the accumulation of gold particles for both used antibodies. Significance of these changes were confirmed by non-parametric, Kruskal-Wallis test (Table 2). A clear reduction in the number of gold particles for both antibodies was observed for most interfaces after prolonged cold treatment (28 h and 7 days) in leaves of CS line, especially for KMS/BS, VP/CC, VP/SET, and CC/SE interfaces (Fig. 5a, b, d–h, Table 2).

**Table 2:**
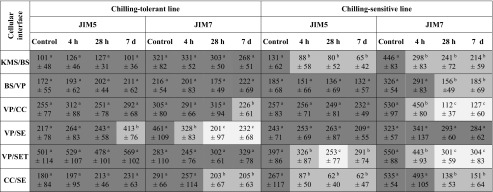
The number (mean ± SD) of gold particles conjugated to monoclonal JIM5 and JIM7 antibodies in the control (non-chilled) and chilled (for 4 h, 28 h, and 7 days) plants of the chilling-tolerant and chilling-sensitive maize lines

Data were collected in three independent experiments. Values for each variant were estimated on the basis of at least 30 cell wall sections (area 1 μm^2^). Value for each chilling treatment was compared with value for control, for each cell interface and maize line separately. Significant differences (*P* < 0.01) estimated by Kruskal-Wallis test with Bonferroni correction are indicated by different superscript letters and shading

*KMS* Kranz mesophyll; *BS* bundle sheath; *VP* vascular parenchyma; *CC* companion cell; *SE* thin-walled sieve tube; *SET* thick-walled sieve tube

### Incipient plasmolysis in cells of maize leaves

**Fig. 6:**
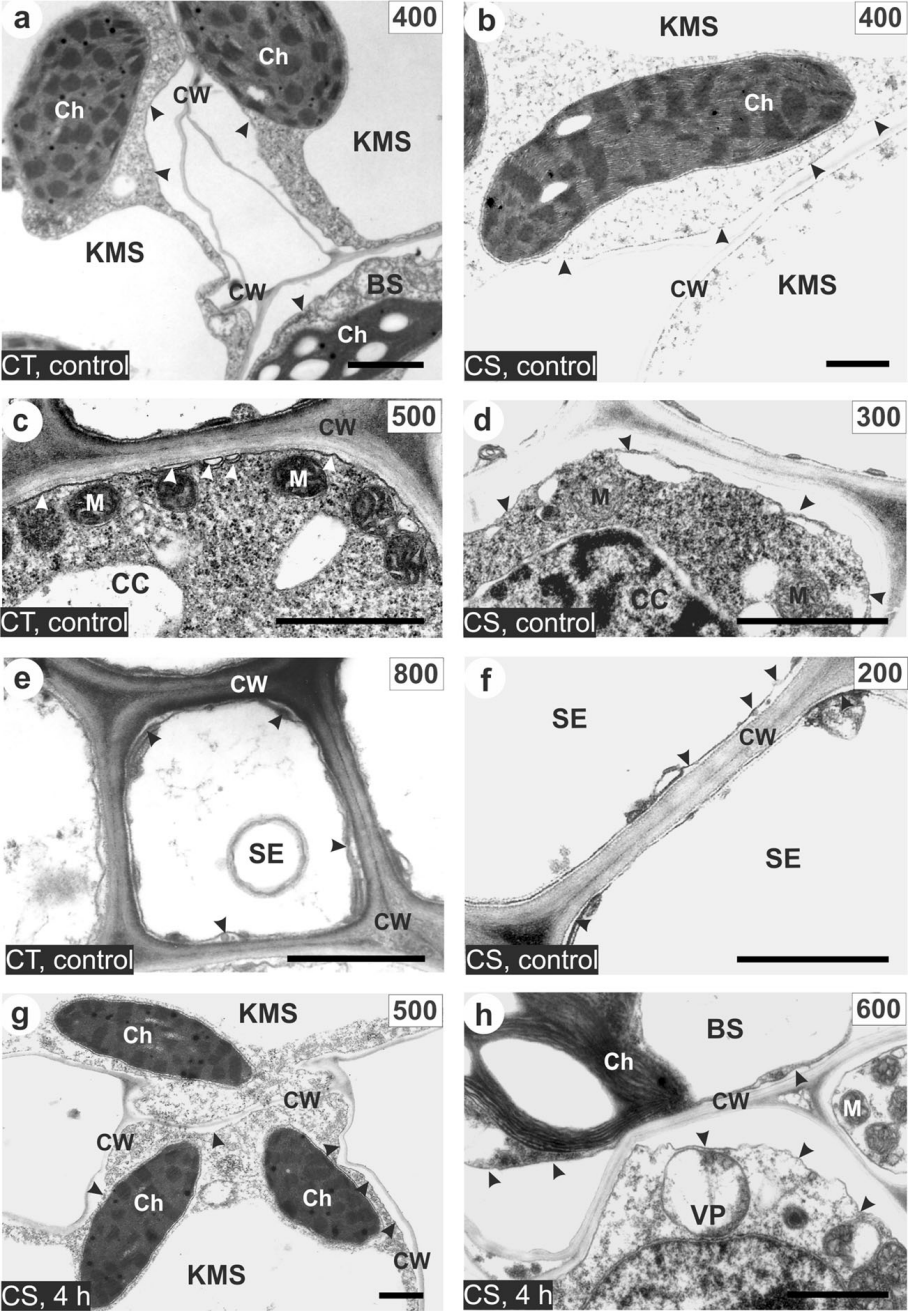
Examples of incipient plasmolysis in cells of control (non-chilled) (**a–f**) and chilled leaves for 4 h (**g**, **h**) of chilling-tolerant (*CT*) and chilling-sensitive (*CS*) maize line incubated in different sorbitol concentration (values in *rectangles*). Note that protoplast is separating from the cell wall (*arrowheads*). *KMS* Kranz mesophyll; *BS* bundle sheath; *VP* vascular parenchyma; *CC* companion cell; *SE* thin-walled sieve tube; *Ch* chloroplast, *M* mitochondria; *CW* cell wall; *Scale bar*, 1 μm

In cells of both maize lines without evidence for plasmolysis the continuity of protoplast adjacency to cell wall was observed. In control plants, KMS and BS cells plasmolysed in approximately the same concentration of sorbitol (Table 3). It was between 300 and 400 mM for the CT and 400 mM sorbitol for CS lines (Table 3; Fig. 6a, b). A strong difference in the osmoticum concentration that led to incipient plasmolysis was found for the CC/SE complex: these cells plasmolysed at a sorbitol concentration of 500 mM in the CC cells and 800 mM in the SE cells of CT line and at 300 or 200 mM in the CS line (Table 3; Fig. 6c–f). Chilling of plants for 1 and 4 h did not distinctly change the osmotic potential of KMS, BS cells, and the CC/SE complex in the CT line (Table 3). The CS line responded to chilling (especially after 4 h) by a strong increase of the concentration of osmoticum needed to cause incipient plasmolysis in KMS and BS cells, while the CC/SE complex remained without significant changes in plasmolysis (Table 3; Fig. 6g, h).

**Table 3:**
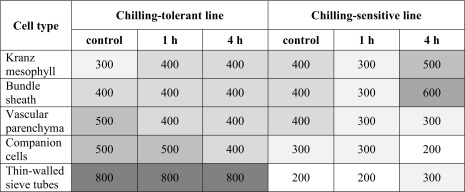
Incipient plasmolysis in leaf cells in two maize inbred lines: chilling-tolerant and chilling-sensitive.

Values represent the concentration of sorbitol solution (in mM), in which the incipient plasmolysis was observed. For improved readability different values were marked by shading. Data were collected in three independent experiments. Each variant was estimated on the basis of at least 10 vascular bundles

### Changes in sucrose accumulation in chilled leaves of maize

**Fig. 7:**
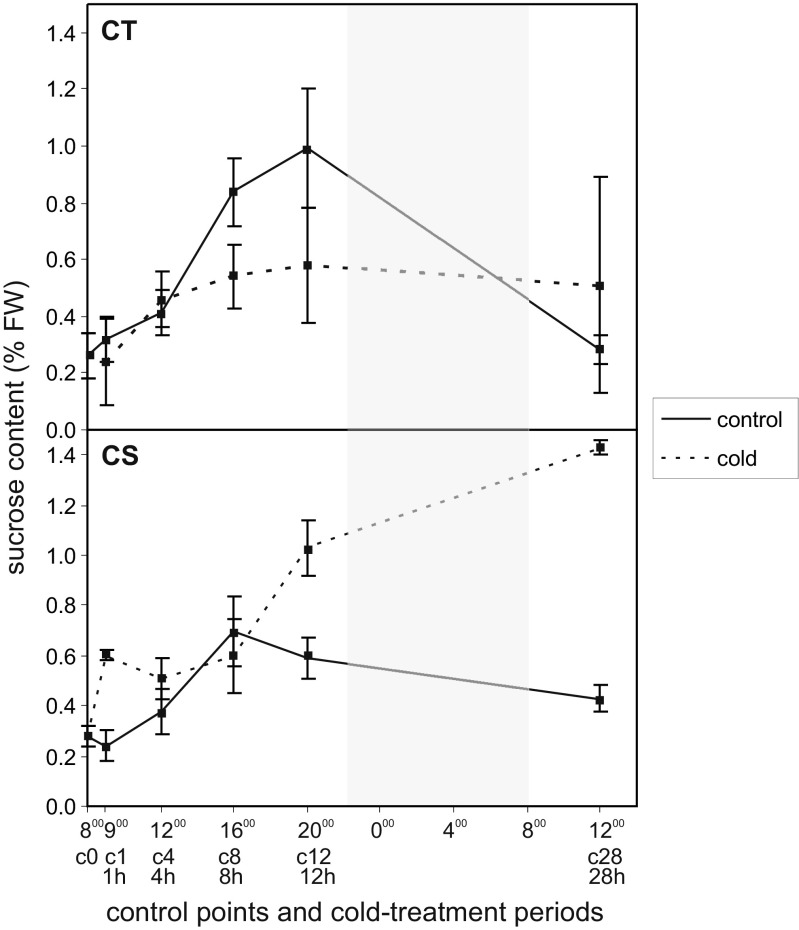
Sucrose content expressed as a percentage of fresh weight (FW) in leaves of two maize lines: chilling-tolerant (*CT*) and chilling-sensitive (*CS*) treated with low temperature for several time periods: 1, 4, 8, 12, and 28 h with corresponding time points for control (non-chilled) plants: c0, c1, c4, c8, c12, and c28. The *shaded area*—period with the light off (night). *Bars* represent the mean ± SD values

Sucrose content in the leaves of control plants of both maize lines corresponded to changes of sugar concentration occurring during the diurnal cycle—at the start of light exposure, the sucrose content was the lowest, and then it increased gradually until the end of exposure, and decreased at dark (Fig. 7). In the chilling-treated plants of CT line, the reduction of sucrose accumulation was observed, wherein, the trend of changes was similar to that obtained for the control plants. However, in CS line, after just the first hour of exposure to low temperature, a drastic increase of the sucrose content was found—the level of sugar was about three times higher compared to its content in leaves of control plants.

## Discussion

In this paper, it was investigated whether changes in the osmotic potential of cells can lead to modification of cell wall properties via the deposition of pectins. The substantial and local changes in the osmoticum seem to be caused by overaccumulation of sucrose in the certain cells probably resulting from the occluded plasmodesmata.

### Are there any changes in cell wall properties under cold conditions?

In this work the properties of the cell wall in the context of global pectin content and PME activity were examined in chilled leaves of two maize inbred lines. CS line was characterized by significantly lower PME activity at cold compared to control variant, what was depended on the duration of chilling treatment of plants (Fig. 3). At the same time, decrease in the content of pectins, particularly after 7 days of cold treatment was observed in leaves of CS line (Fig. 2). The impact of low temperature on the organization of components of the cell wall, including pectins, can be considered in two aspects, as a result of cold stress and as an effect of cold-acclimation process. In the 3-week cold-acclimated winter oilseed rape leaves, significant increase of pectin content was observed (Solecka et al. 2008). It was in accordance with high activity of PME enzyme and changes in biomechanical properties of leaves, e.g., stiffened lamina. In this work the relatively short period of chilling treatment (4 h up to 1 week) of cold-sensitive plants led to the reverse situation which may be the evidence of response of CS maize line to cold stress. This may reflect the necessity of changes in cell wall rigidity for maintaining turgor pressure in particular cells through changes in state of cell wall pectins (Haswell and Verslues 2015). Additionally, a significant reduction of labeling with JIM5 antibodies (which recognize low-esterified HGAs) was observed mainly in the intercellular connections between photosynthetically active cells: KMS and BS cells and CC/SE complex in leaves of CS maize line. The reduction in the level of low-esterified pectins caused by lower PME activity may indicate lower stiffness of the cell wall (Catoire et al. 1998). This loosened structure of the cell wall in CS leaves may be insufficient to maintain a proper turgor pressure, which is necessary in these types of cells and/or whole tissues. This observation also raises the question about the mode and the level of reguation of PME activity in stress conditions. The proteinaceous PME inhibitor (PMEI), ubiquitously expressed in higher plants, may provide an effective post-translational control mechanism for PME activity (Balestrieri et al. 1990; Jolie et al. 2010). PMEIs have revealed specificity toward plant PME only and their physiological function lies in the modulation of the endogenous PME activity during plant growth, development, and stress reactions (Giovane et al. 2004; Jolie et al. 2010). Our data showed that the PME activity in cold differs depending on the genotype of maize line. The secretion of specific PMEIs may modulate enzyme activity depending on their quantity or interplay with PME during plant responses to stress conditions. Searching for the genotype- or tissue-dependent mechanisms responsible for the temperature control of PME activity requires further studies.

### Are pectins specifically localized within the maize cell wall?

In this work, low- and high-esterified homogalacturonans were localized using JIM5 and JIM7 antibodies, respectively, with particular attention to the cell wall around plasmodesmata in the pit fields due to suspected participation of these polysaccharides in the mechanism of plasmodesmata closure at cold as demonstrated earlier (Bilska and Sowiński 2010). However, no relationship between the presence of plasmodesmata and the occurrence of both types of epitopes (detected by JIM5 and JIM7) in maize leaves was found (e.g., Fig. 4f, Fig. 5f). Low-esterified pectins were previously found at the pit fields in the cell walls of tomato (Orfila and Knox 2000) and apple pericarp (Roy et al. 1997). Knox and Benitez-Alfonso (2014) supposed that the composition of cell walls surrounding plasmodesmata significantly differs from other cell wall regions. In this work, for leaves of maize, monocotyledonous plant, the labeling was restricted to areas around plasmodesmata but not in close contact with them. It may give evidence that grass cell wall differs in the local structure and composition in comparison with the cell wall of other plants.

### May modifications of the cell wall properties be associated with cell osmoticum changes?

The studied lines strongly differed in respect to the osmotic pressure of leaf cell, both in control and chilling-treated plants (Table 3). In the CT line, the CC/SE complex plasmolysed at higher concentration of sorbitol by about 300 mM than the KMS, BS, and VP cells (Table 3; Fig. 6a, c, e). This demonstrates a strong solute gradient between these cells and the rest of the leaf cells, caused, as previously noted (Bilska and Sowiński 2010), by an efficient apoplasmic phloem loading of sucrose in CT line. Similar difference in osmotic potential of leaf cells was found by Evert et al. (1978). In contrast to CT line, in control plants of CS line, the CC/SE complex plasmolysed at lower osmoticum concentrations than required to plasmolyse the KMS, BS and VP cells (Table 3; Fig. 6b, d, f). In specific regions of the maize leaf, gradient of the osmotic potential of photosynthetic active cells may be related to the type of phloem loading and ultrastructural differences. Thus, in CS maize line, sucrose transport from mesophyll to vein does not need to undergo against concentration gradient, similarly as in some species showing no apoplasmic phloem loading (Turgeon and Medville 1998). The possibility that active phloem loading (defined as photosynthate transport to the CC/SE complex against the concentration gradient) might not be necessary in some plants was discussed by Turgeon (2006). The observation that osmotic pressure of the CC/SE complex in leaves of CS line seedlings is lower than in adjacent cells is in line with our earlier finding, where the CC/SE complex in leaves of dent type maize lines might not be fully isolated symplasmically from the rest of the leaf (Sowiński et al. 2001, 2003). This might reflect ultrastructural differences in juvenile leaf development between *Z. mays indurata* and *Z. mays indentata* at this physiological stage.

Changes in the osmotic potential of plant cells may occur due to the accumulation of photosynthesis products, especially sucrose with strong osmotic properties (Wolfe 1991). In chilled leaves of CS line, sucrose is accumulated just after 1 h of chilling treatment and remains at high level during the dark (Fig. 7). Hence, it can be assumed that sucrose which was accumulated in KMS, BS, and VP cells could reduce their osmotic potential. The reason for the inhibition of sucrose transport to the CC/SE complex may be rapid changes of plasmodesmata ultrastructure and thus their reduced permeability under cold conditions (Bilska and Sowiński 2010). The most significant differences between tested maize lines were changes in the osmotic potential (Table 3) and the accumulation of sucrose (Fig. 7) after 4 h of chilling treatment. It is known that the osmotic stress induced by NaCl caused changes in the chemical composition of primary cell wall of tobacco (Iraki et al. 1989a, b). These changes, including decrease of soluble pectin content, were accompanied with the inhibition of cell growth. On the other hand, the relationship between the osmotic adjustment and cell expansion may be different in various organs of plants (Westgate and Boyer 1985). For instance, in water-limited conditions the growth of maize silk is more limited than roots (Westgate and Boyer 1985). The maintenance of turgor in cells under water deficit is crucial to provide the appropriate properties of the cell wall, i.e., elasticity. In addition, C_4_ grasses have a better ability of adjusting the osmotic potential than C_3_ grasses, which help them to adapt to water-limited conditions (Barker et al. 1993).

In this work, cell-specific changes of osmotic potential can affect the composition of the cell wall by reducing the global amount of pectins in chilled plants of CS line rather than by the modification of their methylesterification degree. It is possible, that this reduction, at least partially, is related to the activity of another enzyme, polygalacturonase, where its control is dependent on a variety of stress factors, including cold (D’Ovidio et al. 2004; Almeida and Huber 2010; Liu et al. 2014). Additionally, it is possible that by the interaction with the wall-associated protein kinases (WAKs), pectins take part in the signal transduction between cell wall and plasma membrane, and in the control of the distribution of cell wall components (Haswell and Verslues 2015). Then, a relatively rapid change in the osmotic potential, which was observed mainly in the CS line, could be “the starting point” for profound rearrangement of cell wall pectins in this maize line.

## Conclusions

This work shows that low temperature affects the content of cell wall pectins in maize leaves. Local changes in the osmotic potential in leaves of chilled plants of chilling-sensitive maize line, as the result of the rapid accumulation of sucrose (observed after just 1 h of chilling treatment, probably due to the blockage of its transport via cold-modified plasmodesmata) may lead to significant changes in the pectin organization. These changes might be found as indirect effect of cold stress or mechanism of plant adjustment to adverse environmental condition. Our results indicate that this problem is very important for the breeding of chilling-sensitive plants and it is worth pursuing.

## Abbreviations

BS: bundle sheath
CC: companion cell
CS: chilling-sensitive maize line
CT: chilling-tolerant maize line
KMS: kranz mesophyll
PME: pectin methylesterase
PMEI: pectin methylesterase inhibitor
SE: thin-walled sieve tube
SET: thick-walled sieve tube
VP: vascular parenchyma
